# Social isolation increases impulsive choice with minor changes on metabolic function in middle‐aged rats

**DOI:** 10.14814/phy2.70184

**Published:** 2025-01-16

**Authors:** Jassmyn J. Venegas, Jacob M. Weisz, Chan Young Choi, Ren E. Herringshaw, Omar A. Nabelsi, Nu‐Chu Liang

**Affiliations:** ^1^ Department of Psychology University of Illinois Urbana‐Champaign Champaign Illinois USA; ^2^ Division of Nutritional Sciences University of Illinois Urbana‐Champaign Urbana Illinois USA; ^3^ Neuroscience Program University of Illinois Urbana‐Champaign Urbana Illinois USA; ^4^ Carl R. Woese Institute for Genomic Biology University of Illinois‐Urbana Champaign Urbana Illinois USA

**Keywords:** impulsive choice, insulin resistance, metabolic function, middle age, social isolation

## Abstract

The effects of social isolation (SI) during middle age remain unclear, so we tested the hypothesis that SI would lead to an increase in impulsive choice (IC), anxiety‐like behavior, and metabolic dysfunction in middle‐aged rats. Male and female rats were housed individually or in groups of four with same‐sex housing mates at 11 months of age. Two months later, IC behavior was assessed using a delay‐discounting task and anxiety‐like behavior through a novelty‐suppressed feeding (NSF) task. Lastly, glucose tolerance and insulin sensitivity following exposure to a high‐fat diet were assessed using an oral glucose tolerance test (OGTT) and an insulin tolerance test (ITT). The results showed that socially isolated rats displayed more IC behavior than did group‐housed rats of both sexes. However, no significant effect of housing was evident in the NSF task, OGTT, or ITT. Male rats had a higher plasma insulin concentration and insulin resistance index compared to females. Our findings demonstrate that SI in middle age is sufficient to increase IC behavior and highlight inherent sex‐specific differences in metabolic profiles. These findings underscore the importance of investigating mechanisms that underlie the effects of social isolation during different stages of life.

## 1 INTRODUCTION

Human studies show a strong association between social isolation and detrimental health outcomes. Specifically, social isolation is associated with memory loss, impairments in spatial memory, verbal fluency (Lara et al., 2019; Mosen et al., 2022; Read et al., 2020), and an increased risk of developing dementia (Akhter‐Khan et al., 2021; Shen et al., 2022). In addition to cognitive impairments, social isolation is also associated with a higher risk of mortality in the form of increased risk for cardiovascular disease, high blood pressure, and type 2 diabetes (Brinkhues et al., 2017; Christiansen et al., 2021; Xia & Li, 2018). In humans, the incidence of social isolation appears to increase with age. One study examining the rate of social isolation found that 5.4% of individuals aged 18–39 reported feeling socially isolated. This percentage increased to 12.7% among those aged 40–50 and to 17.9% among individuals aged 50–59 (Röhr et al., 2021). Moreover, people who experience social isolation later in life are up to 40% more likely to develop dementia (Sutin et al., 2020). Due to the increased perception and incidence of social isolation in older adults, it is imperative to further understand the effect of social isolation on cognitive and metabolic function in this population.

In rodent studies, social isolation is typically implemented early in development. Rats reared in social isolation from postnatal day 23–25 for 1 month exhibited decreased working memory performance on a radial arm maze, as evidenced by an increase in total errors compared to pair‐ or group‐housed rats raised in an enriched environment (Juraska et al., 1984; Seymoure et al., 1996). Additionally, rats socially isolated for 3 months from 4 weeks of age display impaired short‐term memory in passive avoidance tests and increased depression‐ and anxiety‐like behaviors compared to group‐housed rats (Song et al., 2021). In adult mice, 3 weeks of social isolation also leads to an increase in anxiety‐like behavior in an open field test and elevated plus maze (Berry et al., 2012). Impulsive behavior has also been shown to be influenced by social isolation. Human social isolation can lead to an increased risk of making impulsive decisions and aggressive behaviors (Otten & Jonas, 2013). In animal models, impulsivity is often studied through an impulsive choice (IC) and/or impulsive action task. The assessment of IC involves a delay‐discounting task in which the subject's preference for a larger, later reward versus an immediate, smaller reward is examined. An increased preference for the immediate, smaller reward would indicate IC behavior (Hamilton et al., 2015). A study investigating the effects of social isolation beginning on postnatal day 22 and lasting for 9 weeks found that isolated rats exhibited a decrease in instrumental learning, an increase in impulsive action, and no difference in IC compared to socially housed rats (Baarendse et al., 2013). However, more research is needed to elucidate the relationship between social isolation and impulsivity across age, particularly in middle‐aged and aging adults.

Although there is a strong association between social isolation and an increased risk of type 2 diabetes in humans, few rodent studies addressed this issue. A recent study found that male rats subjected to 7 weeks of social isolation beginning on postnatal day 21 have an increased homeostatic model assessment for insulin resistance (HOMA‐IR) value, which indicates higher insulin resistance via measures of fasting blood glucose and insulin levels (Bove et al., 2022). Such findings suggest that social isolation may directly impair insulin signaling and glucose metabolism while indicating the need to examine the effects of social isolation on metabolic function in different age populations. Further examination of the influence of social isolation on glucose homeostasis and insulin sensitivity is essential to understanding the potential role of social isolation in the development of type 2 diabetes.

Most research on social isolation investigates its effects on cognitive function, predominantly in male subjects during the post‐weaning stage, adolescence, or young adulthood. (Lapiz et al., 2003; Shao et al., 2013; Song et al., 2021; Zorzo et al., 2019). To date, few studies address potential sex differences on the influences of social isolation, and no studies have assessed the effects of social isolation in male and female older rats. One study found that male rats experiencing daily bouts of social isolation from postnatal day 15–21, followed by pair housing, displayed greater working memory impairments on a radial maze task compared to their female counterparts at 3 months of age (Sandstrom, 2005). A separate study found that although social isolation starting at ~54 days impaired information processing similarly in both male and female rats, socially housed males outperformed socially housed females (Elliott & Grunberg, 2005). Taken together, these findings suggest male rats may be more susceptible to the effects of social isolation on working memory and more responsive to the effects of social enrichment compared to female rats as male socially enriched rats have greater information processing compared to female socially enriched rats. However, it is important to note that a separate study found that male and female rats reared in an enriched or socially isolated environment perform comparably on a radial arm maze task (Juraska et al., 1984). Thereby highlighting the need for more studies to examine the role of sex in social isolation in a variety of physiological and behavioral measures.

Given the significant role of the social environment in cognitive and metabolic functions and the lack of studies assessing the effect of social isolation on these functions in middle‐aged rats, the present study aims to address this gap. We socially isolated or group‐housed middle‐aged, approximately 11‐month‐old, male and female rats for 2 months and subsequently assessed their cognitive performance and metabolic function. The 2‐month isolation aligns with previous studies that typically use isolation periods of 30–90 days (Arakawa, 2018). Although studies have shown increased anxiety‐like behavior after shorter durations, previously group‐housed, middle‐aged rats may not be as susceptible to isolation as younger rats. Importantly, this extended isolation period is intended to mimic prolonged social isolation in humans, such as extended periods of reduced social contact or minimal interactions that can be seen in middle‐aged individuals due to life changes or loss of social networks. We tested the hypothesis that socially isolated middle‐aged rats would exhibit more impairments in cognitive, executive, as well as metabolic function through performance in memory and IC tasks, a variety of anxiety‐like behavior tests, and glucose tolerance and insulin sensitivity tests.

## 2 MATERIALS AND METHODS

### 2.1 Subjects

Subjects included 22 male and 22 female Sprague–Dawley retired breeder rats (Envigo, Indianapolis, IN) that had been socially housed with the same sex at the vendor's facility and were ~11 months old when they arrived. Upon arrival, all rats were housed in same‐sex groups of four per cage and kept on a 12:12 reverse light/dark cycle (lights on at 14:00 h) in a temperature‐controlled room. Nine days after arrival, rats were divided into four groups. Group sizes were determined through a power analysis (OriginPro, power >0.85, alpha 0.01), observed power from previous studies, and literature reports using similar methods (Yang et al., 2022). Male and female group‐housed rats were housed as four rats per cage in a transparent breeder cage with the dimension of 45.5 × 35 × 17.8 cm (M‐GH and F‐GH, *n* = 12/sex). Male and female socially isolated rats were housed individually in a transparent standard‐size cage with the dimension of 45.5 × 23.6 × 17.8 cm (M‐SI and F‐SI, *n* = 10/sex). Rats were assigned to groups in a semi‐randomized manner, taking body weight into account to ensure similar average weights between groups within each sex. This approach minimized any potential body weight differences across the groups upon initiation of the study. Average body weights across groups over the course of the study are summarized in Table 1. All rats were handled 5 days a week to measure body weight and during scheduled cage cleaning events and estrous cycle in female rats were not monitored to ensure minimized daily handling. Altogether, handling was kept to an absolute minimum.

**TABLE 1:** Body weights across housing conditions.

Group	Body weight (g)		
	Initial	2‐month	Final
Female group housed	299.81 ± 5.12	313.90 ± 6.53	340.22 ± 7.88
Female socially isolated	309.66 ± 4.86	308.69 ± 6.28	324.05 ± 6.83
Male group housed	487.57 ± 16.71	517.65 ± 18.79	558.30 ± 19.04
Male socially isolated	498.24 ± 16.16	526.40 ± 14.82	545.98 ± 16.20

**FIGURE 1:**
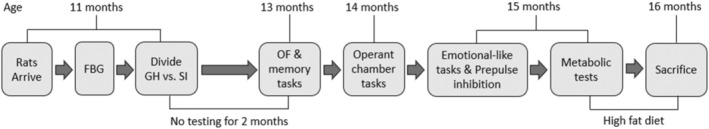
Experimental timeline. The timeline illustrates all tasks conducted during the experiment. Nine days after arrival, rats were socially isolated (SI) or group housed (GH) and remained in these conditions until the conclusion of the study. Results reported herein include impulsive choice measured in the operant chambers, novelty suppressed feeding (NSF) as one of emotional‐like tasks, and oral glucose and insulin tolerance as metabolic tests. Negative findings from the fasting blood glucose (FBG) measurement, open field (OF) test, memory tasks, prepulse inhibition assessment, and other emotional‐like behavior assessments are detailed in Appendix S1.

Rats were kept in their assigned housing conditions until the end of the study. A summary of the experimental timeline is presented in Figure 1. In addition to the tasks discussed here, further behavioral and metabolic assessments, as well as body fat composition analyses, are detailed in Appendix S1. The open field (OF) and memory tasks, including the novel object recognition, object location task, and Barnes maze, were conducted before the IC task. Following the IC task, the rats' depression‐like behavior was assessed through a sucrose preference test, after which their sensorimotor gating function was assessed using a prepulse inhibition task. These assessments, which also include high‐fat diet choice, fasting blood glucose measurements, and sacrifice blood glucose readings, showed no significant effects of housing condition. Therefore, information of these tasks is included in Appendix S1. This report focuses on results from the IC task, NSF task, and oral glucose and insulin tolerance tests. Rats received ad libitum access to water and standard chow (3.1 kcal/g; Envigo Teklad 18% protein diet) for ~ three‐months, after which rats underwent IC assessment. Four days prior to the oral glucose tolerance test (OGTT), the standard chow diet was replaced with a Western diet (4.7 kcal/g; 43% carbohydrates, 40% fat, 17% protein; D12079B, Research Diets, New Brunswick, NJ). During video scoring of tasks such as the NSF, scorers were not aware of the group assignment for each rat, allowing for blinding in the data acquisition process. All procedures were approved by the Institutional Animal Care and Use Committee at the University of Illinois, Urbana‐Champaign (Protocol # 19253) and are in accordance with the Guide for the Care and Use of Laboratory Animals.

### 2.2 Impulsive choice

Rats were food restricted and maintained at ~85% of their free‐feeding weight until the completion of the IC, fixed ratio, and progressive ratio assessment. IC was evaluated in operant chambers utilizing a delay‐discounting task, as previously described and published by our laboratory (Yang et al., 2022). Training and testing occurred during the dark cycle, beginning 5 h before light onset (07:00 h) and ending 1 h before light onset (13:00 h). Assignments of the levers were counterbalanced such that 50% of rats received the smaller immediate reward from the left lever and the larger reward from the right lever. The other 50% of rats had the opposite lever assignment. Lever assignments were held constant throughout the IC assessment. Rats underwent 1 day of magazine training (MT) where they learned that pellets would be dispensed into the dish inside the chamber. Following MT, rats underwent 6 days of lever press training where they learned to associate lever pressing with receiving the corresponding reward. During lever press training, if the rat was not associating pressing the lever with receiving a reward, it would undergo a separate lever press training during the light cycle (13:00 h) where hand‐shaping would occur. Hand shaping consisted of the researcher gently guiding the rat's paw to press the lever and then directing it to the dish where the pellet was dispensed. Following lever press training, the rats underwent 2 days of forced choice training to learn to press both levers. During this training, both levers were extended at the start of the first trial. After a rat pressed a lever (either the right or left) and received their reward, the session would enter the inter‐trial interval (ITI). In the next trial, only the lever that was not pressed in the previous trial was extended. In the subsequent trial, both levers were again presented.

Upon completion of forced‐choice training, rats underwent 2 days of pre‐training. Pre‐training sessions ended after 45 min or 45 trials, whichever came first. During these sessions, both levers were extended, and cue lights above both levers were on. Once a lever was pressed, the cue lights turned off, and the corresponding pellets were delivered after 1 s. After pellet delivery, the house light turned on for 20 s, this was the ITI. After the ITI, the house light turned off and a new trial began. Following pre‐training, rats underwent discrimination training. The only alteration between discrimination and pre‐training was that an omission error was added, in which the levers would retract if a rat did not press a lever within 60 s, and the trial would move onto the ITI without dispensing a reward. Discrimination training ended once the rat chose the larger delayed lever at least 80% of the time for three consecutive days.

**FIGURE 2:**
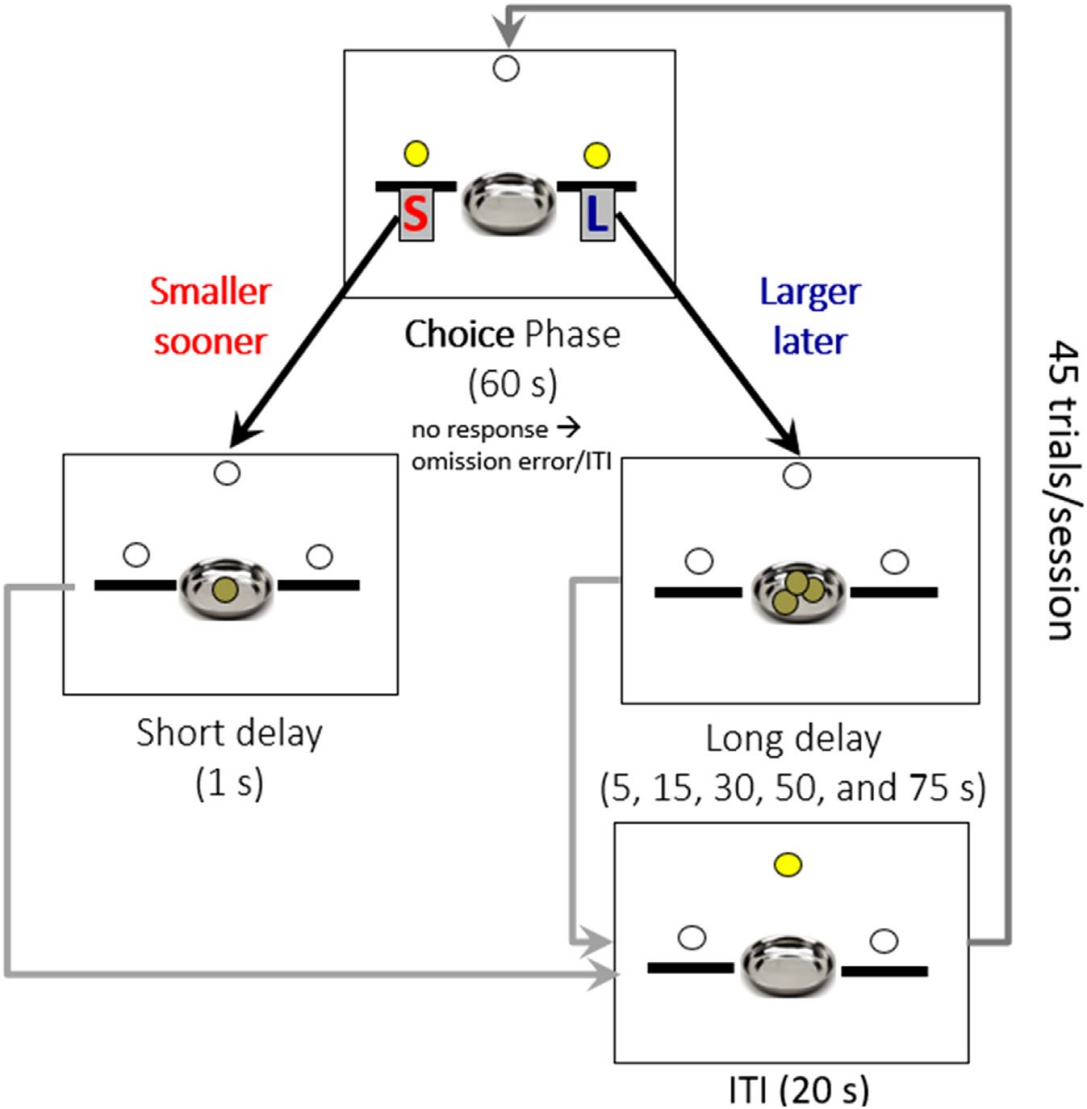
Summary of the impulsive choice tests. Each trial begins with a choice phase where the rat responds to ether the lever with a short 1 s delay or a long delay to receive the respective reward. Throughout the 5 days of testing, a press on the lever with a short delay always renders delivery of 1 food pellet after a 1 second delay. By contrast, the delay to receive 3 food pellets when the rat chooses to press on the lever with long delay increases from 5 s on the first testing day to 15, 30, 50, and finally to 75 s on the second, third, fourth, and fifth day of testing, respectively. Each choice trial is followed by an inter‐trial interval (ITI) of 20 s. An omission is recorded if the rat does not make a response within 60 s, and the trial proceeds directly to the ITI. On each testing day, the session ends once the rat completes 45 trials.

Testing began once the above‐mentioned criteria were met. IC was assessed over the course of 5 days by adding a delay to the larger, delayed reward lever. The delay of reward delivery for the larger reward lever maintained constant within each testing day but was increased from 5, 15, 30, 50, to 75 s throughout the five‐day testing period, whereas the smaller, immediate reward lever always dispensed 1 pellet after 1 s (Figure 2). Testing procedures were identical to the discrimination training. Rats progressed to the next day of testing regardless of performance. IC assesses impulsive decision‐making by examining the tendency to prefer a smaller, immediate reward over a larger, delayed reward (Yang et al., 2022). To examine whether the rats were making more impulsive choices as the delay increased, the preference ratio for the larger reward lever was calculated for each delay interval to indicate IC behavior. A score of one indicates the rat pressed the larger reward lever for all the trials it completed during that testing day and suggests no IC behavior.

### 2.3 Fixed ratio

The day following the conclusion of IC testing, where the longer delayed lever dispensed 3 pellets after a 75‐s delay, rats underwent a fixed ratio session using an FR1 schedule on their designated smaller, sooner lever to assess their rate of responding. This session lasted for a total of 30 min, during which the total number of lever presses was recorded.

### 2.4 Progressive ratio

The day after completing the fixed ratio schedule of reinforcement, rats participated in a progressive ratio task to assess their motivation to obtain the reward. Rats were placed in the operant chamber and placed on a PR2 schedule on their assigned smaller sooner lever. The session ended after 30 min or after 5 min passed without the rat completing the necessary lever presses to obtain a pellet reward. The breakpoint, representing the total lever presses the rat is willing to make to obtain the reward, was recorded.

### 2.5 Novelty suppressed feeding

All food hoppers were removed from the home cages 24 h before testing, and four Cheerios per male and three Cheerios per female were provided in their home cages. On the testing day, rats were weighed, individually placed in standard cages, and then transferred to the testing room. They were given 30 min to habituate. Testing began 2 h after light onset. Each rat underwent one 5‐min testing session, which was video recorded through a camera anchored in the ceiling of the testing room. During this session, a rat was placed in the corner of the center outlined square (23.5 × 23.5″) within the larger open field arena (44 × 44″), where Honey Nut Cheerios (General Mills, Inc) were positioned in the center. Once the 5 min elapsed, the rat was placed back into their holding cage, and the number of Cheerios eaten was recorded. The arena was disinfected between rats, and fresh cheerios were provided per each session. One viewer watched the videos, identified when the rat began chewing the Cheerio, and recorded the time as latency to eat during the NSF test.

### 2.6 Oral glucose tolerance test

To provide a metabolic challenge in which differences in glucose metabolism could be observed, an OGTT was performed following a five‐day exposure to a Western diet (4.7 kcal/g; 43% carbohydrates, 40% fat, 17% protein; D12079B, Research Diets, New Brunswick, NJ). This short high‐fat diet feeding period was chosen to introduce an acute metabolic challenge to assess potential differences in metabolic processes across groups. Previous studies have demonstrated that a 7‐day exposure to HFD is sufficient to induce changes in metabolic responses (Swartz et al., 2010). The day prior to the OGTT, all rats were orally gavaged with a bolus of water to habituate them to the gavage procedure. On the day of the OGTT, rats were removed from their home cages 8 h prior to testing and housed individually in standard cages without food. Rats were left to habituate in the testing room for 30 min. Testing began 2 h after light onset. After measuring fasting blood glucose levels using an AlphaTRAK2 glucometer and tail blood collection at 0 min, rats were orally gavaged with a 20% bolus of glucose (2 g/kg). Additional tail blood collection and glucose readings were measured 20, 60, and 120 min after the oral gavage of glucose. Following testing, rats were kept in their holding cages for 15 min to allow for further blood clotting from the tail wound before returning to their home cages. Insulin concentration from the plasma collected during the OGTT was measured using the Rat Ultrasensitive Insulin ELISA kit, in accordance with the manufacturer's protocol (ALPICO, Salem, NH; Catalog No. 80‐INSRTU‐E01).

Blood glucose readings and plasma insulin measured during the OGTT were used to assess insulin resistance using the HOMA‐IR (Matthews et al., 1985). Peripheral insulin sensitivity was assessed using the insulin sensitivity index (ISI_0,120_) (Gutt et al., 2000). The formula for each surrogate index is listed below.

$$\text{HOMA}-\mathrm{IR}=\frac{\text{fasting insulin}\;\left(\frac{\mathrm{\mu U}}{\mathrm{ml}}\right)\times \text{fasting glucose}\;\left(\frac{\text{mmol}}{L}\right)\;}{22.5}$$

$${\mathrm{ISI}}_{0,120}=\frac{\text{glucose load}\;\left(\mathrm{mg}\right)+\left({\text{glucose}}_{0\min}-{\text{glucose}}_{120\min}\left(\frac{\mathrm{mg}}{L}\right)\right)\times 0.19\text{body weight}\;\left(\mathrm{kg}\right)}{120\times \log\left(\frac{{\text{insulin}}_{0\min}-{\text{insulin}}_{120\min}\left(\frac{\mathrm{mU}}{L}\right)}{2}\right)+\left(\frac{{\text{glucose}}_{0\min}-{\text{glucose}}_{120\min}\left(\frac{\text{mmol}}{L}\right)}{2}\right)}$$

### 2.7 Insulin tolerance test

Three days after the OGTT and 8 days after Western diet exposure, rats underwent an ITT. Five hours prior to testing, rats were individually housed in holding cages without food. Rats were moved to the testing room and allowed to habituate for 30 min. Testing began approximately 2 h after light onset. After the fasting blood glucose reading (at 0 min), rats were given an intraperitoneal (IP) injection of 1 U/mL of diluted Humulin (Humulin R U‐100, Lilly USA, Indianapolis, IN). Subsequent blood glucose readings were taken 30, 90, and 240 min following the injection. Rats were returned to their home cages 15 min after the last blood glucose measurement.

### 2.8 Data analysis

Statistical analysis was performed in RStudio (version 4.2.1). The preference ratio for IC was calculated by dividing total longer delayed lever presses by total lever presses for each delay. A three‐way mixed model ANOVA was used to analyze preference ratio data, with delay (1–75 s) as a within‐subjects factor and sex (male vs. female) and housing (socially isolated vs. group housed) as between‐subject factors. Data from oral glucose tolerance and insulin tolerance tests were analyzed separately using a three‐way mixed model ANOVA, with time as the within‐subject factor and sex and housing as between‐subject factors. A Tukey's HSD post hoc analysis was conducted for significant results. If the assumption of sphericity was violated, a Greenhouse–Geisser correction was applied to reduce the risk of a type 1 error. The remaining data was analyzed separately using two‐way ANOVAs if normality and homogeneity of variance assumptions were met, with sex and housing as between‐subject factors. Tukey's HSD post hoc analysis was conducted for between‐group differences when a main effect was revealed. For cases where assumptions of the two‐way ANOVA were violated and no interaction effects were observed, the Kruskal–Wallis non‐parametric test was applied. Separate Kruskal–Wallis tests were conducted for housing and sex. Because ANOVAs rely on SEM, presenting data as mean ± SEM aligns with the method of statistical analyses. Due to health complications, a group‐housed female rat was sacrificed following the operant chamber tasks, and data obtained from this rat was therefore not present in subsequent analyses.

## 3 RESULTS

### 3.1 Impulsive choice

**FIGURE 3:**
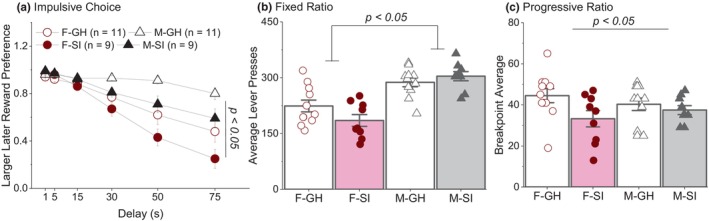
Results of the instrumental tasks. (a) Compared to their isolated (SI) counterparts, group housed (GH) rats had a higher preference for the larger reward. Overall, females decreased their preference for the larger reward to a greater extent than males. (b) Males made significantly more lever presses compared to females. (c) GH rats had higher break points than SI rats.

A three‐way mixed model ANOVA was conducted on the preference ratio for the larger reward lever across delay. Four rats (one from each group) were excluded from the analysis: two failed to pass the discrimination phase, and two experienced chamber malfunctions during testing. Due to the assumption of sphericity being violated, a Greenhouse–Geisser correction was applied. There was no significant interaction effect between sex, housing, and delay on preference for the larger, later reward [*F*(1.77, 63.8) = 0.07, *p* = 0.92; Figure 3a]. A significant main effect of housing was observed [*F*(1, 36) = 7.26, *p* = 0.01], with group‐housed rats exhibiting a stronger preference for the larger, delayed reward compared to socially isolated rats. Additionally, a significant main effect of sex was found [*F*(1, 36) = 21.62, *p* < 0.001], indicating that males had a stronger preference for the larger, delayed reward compared to females. Significant interactions were detected between housing and delay [*F*(1.77, 63.8) = 8.79, *p* < 0.001], and between sex and delay [*F*(1.77, 63.8) = 13.08, *p* < 0.001]. Post hoc analysis revealed that male rats exhibited a stronger preference for the larger reward at the 50 and 75 s delays (post hoc *p* < 0.05) than did females. Although the interaction between housing and delay was significant, the post hoc analysis did not reveal significant differences between the housing conditions (grouped vs. isolated) within a specific delay.

### 3.2 Fixed ratio

The rats excluded from the IC analysis were also omitted from the fixed ratio and progressive ratio analysis. A two‐way ANOVA revealed no main effect of housing [*F*(1, 36) = 0.61, *p* = 0.44; Figure 3b] or interaction effects [*F*(1, 36) = 3.79, *p* = 0.06] on total lever presses. However, there was a main effect of sex [*F*(1, 36) = 39.16, *p* < 0.001]; males made more lever presses than females.

### 3.3 Progressive ratio

A break point represents the maximum number of lever presses that the rat is willing to perform to receive the reward during the session of a progressive ratio task. The two‐way ANOVA revealed no significant main effect of sex [*F*(1, 36) = 0.02, *p* = 0.90] or interaction effects [*F*(1, 36) = 1.66, *p* = 0.21]. However, there was a significant housing effect [*F*(1, 36) = 4.66, *p* = 0.04]; socially isolated rats exhibited lower break points compared to group‐housed rats, an effect that seems to be driven primarily by a difference between F‐GH and F‐SI rats (post hoc, *p* = 0.08, Figure 3c).

### 3.4 Novelty suppressed feeding

**FIGURE 4:**
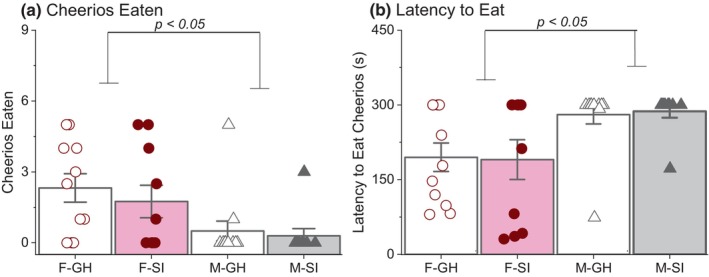
Results of novelty suppressed feeding. (a) Regardless of housing, females consumed more Cheerios than males. (b) Females started to consume Cheerios sooner than males. A score of 300 s was recorded if the rat did not eat a Cheerio.

Two separate Kruskal‐Wallis tests were conducted to assess the effect of sex or housing on Cheerios eaten. There was no housing effect [H(1) = 0.33, *p* = 0.57; Figure 4a], but females ate more Cheerios than males [sex effect: H(1) = 8.60, *p* = 0.003]. Two additional Kruskal–Wallis test were conducted to assess the effect of sex or housing on latency to eat Cheerios. Similarly, there was no housing effect [H(1) = 0.07, *p* = 0.80; Figure 4b], but females were quicker to eat Cheerios compared to males [sex effect on latency to eat: H(1) = 9.25, *p* = 0.002].

### 3.5 Oral glucose tolerance test non‐normalized data

**FIGURE 5:**
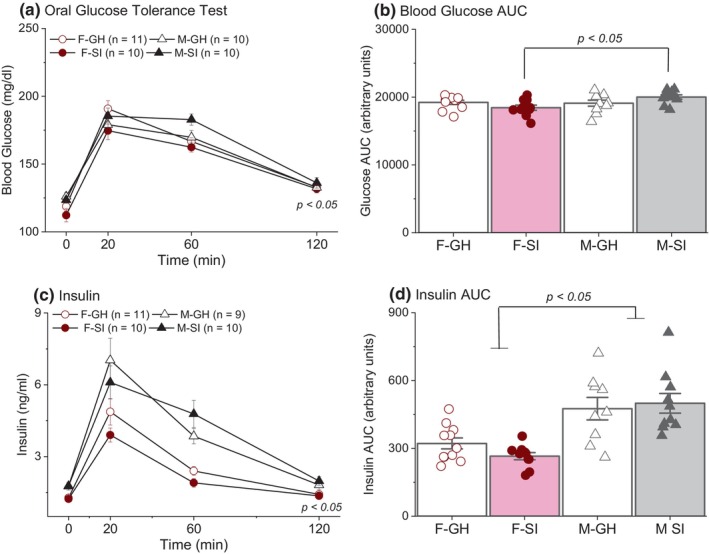
Results of the oral glucose tolerance test. (a) Female socially isolated (F‐SI) rats had lower blood glucose levels compared to male socially isolated (M‐SI) and group housed (M‐GH), and female group housed (F‐GH) rats. (b) F‐SI rats exhibited a lower AUC compared to M‐SI rats. (c, d) Male rats had higher insulin levels and a higher insulin AUC compared to females.

Data from two group‐housed male rats were excluded from data analysis: one due to lack of individual housing for food deprivation prior to the OGTT and the other due to a failed oral gavage. A Greenhouse–Geisser correction was applied because of a sphericity assumption violation. A three‐way mixed model ANOVA of non‐normalized blood glucose level revealed no significant main effect of sex [*F*(1, 37) = 4.12, *p* = 0.05; Figure 5a] or housing [*F*(1, 37) = 0.12, *p* = 0.73]. As expected, there was a significant main effect of time [*F*(2.22, 82.21) = 244.69, *p* < 0.001]. There was a sex by housing interaction [*F*(1, 37) = 4.72, *p* = 0.04], such that female socially isolated rats had lower blood glucose levels compared to their group‐housed female and male counterparts (post hoc *p* < 0.05). Interestingly, females had higher fasting blood glucose levels than males at 11 months of age (see Figure S2A; post hoc *p* < 0.05). This higher level of fasting blood glucose prior to any experimental manipulation may result from a decline in estrogen levels during middle age, as decreased estrogen levels have been associated with insulin resistance (Mauvais‐Jarvis et al., 2013). Since this baseline level of fasting blood glucose was measured 6 days after arrival to our facility, the higher levels in females may reflect a slower recovery from the stress of shipping compared to males. After being housed in our facility for >3 months and a 4‐day exposure to a high‐fat diet, however, males had higher fasting blood glucose levels measured at the 0 min time point during the OGTT compared to females [*F*(1, 39) = 7.04, *p* = 0.01].

Area under the curve (AUC) of blood glucose was used as an index of overall glucose clearance during the OGTT. A lower AUC indicates a better ability to clear glucose from the blood. A two‐way ANOVA on the results of the blood glucose AUC revealed no significant main effect of sex [*F*(1, 37) = 3.79, *p* = 0.06; Figure 5b] or housing [*F*(1, 37) = 0.01, *p* = 0.91]. The interaction effect was significant [*F*(1, 37) = 5.57, *p* = 0.02], revealing that female socially isolated rats exhibited a better blood glucose clearance compared to male socially isolated rats (post hoc, *p* < 0.05).

Data from the same two group‐housed males excluded from the blood glucose OGTT analysis and an additional group‐housed male with an insulin concentration too high to be assessed by the ELISA kit at the 20‐min time point were excluded from the analysis of plasma insulin concentration. The three‐way ANOVA analysis revealed no significant main effect of housing [*F*(1, 36) = 0.54, *p* = 0.47; Figure 5c]. There was a significant main effect of sex [*F*(1, 36) = 28.26, *p* < 0.001] such that males had higher plasma insulin concentrations than females. To evaluate overall insulin levels, insulin AUC during the OGTT was calculated. A 2‐way ANOVA revealed that insulin levels during the OGTT were higher in males than females [*F*(1, 36) = 30.73, *p* < 0.001].

### 3.6 Oral glucose tolerance test normalized data

To account for baseline differences during the OGTT, a separate three‐way mixed‐model ANOVA was conducted on data normalized to blood glucose levels prior to the oral gavage, for example, the 0‐min time point. This analysis revealed a significant main effect of sex [*F*(1, 37) = 4.94, *p* = 0.03] such that females had a higher magnitude of increase in blood glucose during the OGTT. However, the effect of housing was not significant [*F*(1, 37) = 3.06, *p* = 0.89]. The main effect of time was significant [*F*(2.1, 77.88) = 216.23, *p* < 0.001], whereas the sex by housing interaction was not [*F*(1, 37) = 1.59, *p* = 0.22].

A two‐way ANOVA on the AUC of normalized blood glucose revealed significant main effects of sex [*F*(1, 37) = 4.21, *p* = 0.047] and housing [*F*(1, 37) = 4.35, *p* = 0.04], indicating that females had a higher AUC than males, and isolated rats had a higher AUC compared to group‐housed rats. This effect appears to be primarily driven by the lower blood glucose AUC in male group‐housed rats. The interaction effect was not significant [*F*(1, 37) = 1.85, *p* = 0.18].

### 3.7 Insulin sensitivity

Two separate 2‐way ANOVAs revealed no effect of housing or interaction effect of sex by housing on HOMA‐IR or ISI_0,120_ (Table 2). However, there was an effect of sex with males showing a higher HOMA‐IR index [*F*(1, 36) = 19.609, *p* < 0.001] and lower ISI_0,120_ [*F*(1, 36) = 6.06, *p* < 0.001] compared to females, suggesting reduced insulin sensitivity in male rats.

**TABLE 2:** Insulin sensitivity.

Group	HOMA‐IR	ISI_0,120_
Female group housed	9.24 ± 0.65^a^	0.53 ± 0.01^a^
Female socially isolated	8.66 ± 1.00^a^	0.55 ± 0.02^a^
Male group housed	14.49 ± 1.49	0.50 ± 0.02
Male socially isolated	13.62 ± 1.15	0.50 ± 0.01

*Note*: Data are represented as the mean ± SEM.

Abbreviations: HOMA‐IR, Homeostatic assessment of insulin resistance; ISI_0,120_, Gutt's Insulin sensitivity index.

^a^ Females vs. males, *p* < 0.05.

### 3.8 Insulin tolerance test

**FIGURE 6:**
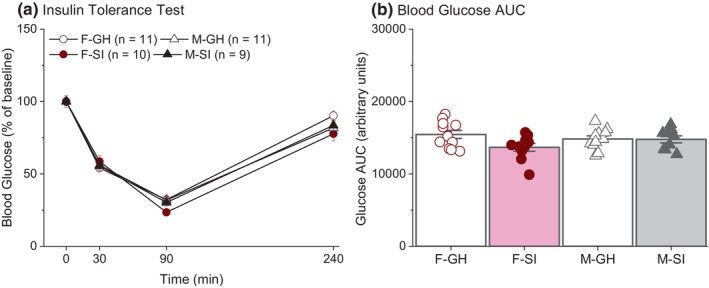
Results of the insulin tolerance test. (a) All rats showed a reduction in blood glucose levels 30 min after a Humulin injection. (b) No significant differences in the AUC of normalized blood glucose levels.

To account for baseline differences in blood glucose levels, a three‐way mixed model ANOVA was conducted on data normalized to baseline blood glucose levels. Data from two male rats, one group housed and one socially isolated, were excluded from the analysis because of inadequate Humulin injection. There were no sex [*F*(1, 37) = 0.03, *p* = 0.87; Figure 6a], housing [*F*(1, 37) = 1.11, *p* = 0.30], or interaction [*F*(1, 37) = 1.03, *p* = 0.32] effects. All rats exhibited comparable fluctuation in normalized blood glucose levels across time. AUC was also analyzed using the normalized data. The AUC calculated from the baseline time point (0 min) to the last time point (240 min) was used as an index of the counterregulatory response to hypoglycemia. A higher AUC value indicates a better counterregulatory response, reflecting the ability of blood glucose levels to rise after a hypoglycemic episode. A two‐way ANOVA on the AUC revealed no housing [*F*(1, 37) = 3.50, *p* = 0.07; Figure 6b], sex [*F*(1, 37) = 0.15, *p* = 0.70], or interaction effects [*F*(1, 37) = 2.97, *p* = 0.09]. Although the normalized data did not show statistical differences across groups, analysis using the raw blood glucose levels during ITT revealed significant effects of sex and the interaction between sex and housing conditions. Specifically, a three‐way mixed model ANOVA revealed that females had lower blood glucose levels compared to males [*F*(1, 37) = 14.73, *p* < 0.005], and F‐SI rats had lower blood glucose levels compared to F‐GH rats at the 90‐ and 240‐min time points (post hoc *p* < 0.05), indicating a potential delay in the counterregulatory mechanisms to hypoglycemia in F‐SI rats. This effect was further supported by the results of the two‐way ANOVA on glucose AUC, which revealed that F‐SI rats had significantly lower blood glucose AUC compared to all other groups (post hoc *p* < 0.05; see Figure S9B).

### 3.9 Summary of results of statistical analyses

Detailed statistical results from other behavioral and additional body composition assessments, which revealed no effect of housing, can be found in Appendix S1. Although no significant housing effect was found in these assessments, some exhibited a significant effect of sex (Tables 3 and 4). At baseline, before the experimental procedures began, females had higher fasting blood glucose levels compared to males (Figure S2A). In the open field test, males spent more time in the center compared to females (Figure S3A). Females had a higher discrimination index in the novel object recognition task (Figure S4B). In the prepulse inhibition test, females exhibited a lower startle response compared to males (Figure S7A). Regarding weight and fat distribution, males had a higher percent weight gain over the 2‐month period (Figure S2B), even though females had more gonadal and mesenteric fat. Males had greater retroperitoneal fat compared to females (Figure S10).

**TABLE 3:** Effect of sex and housing on behavioral tasks.

Task	Measure	Sex effect	Housing effect
Open field	Time in the center	*p <* 0.01 Males > Females	ns (*p* = 0.18)
	Center entries	ns (*p* = 0.47)	ns (*p* = 0.60)
Object location	Discrimination index	ns (*p* = 0.10)	ns (*p* = 0.91)
Novel object recognition	Discrimination index	*p* = 0.04 Males < Females	ns (*p* = 0.90)
Barnes maze	Latency	ns (*p* = 0.10)	ns (*p* = 0.97)
	Error	ns (*p* = 0.60)	ns (*p* = 0.06)
1% Sucrose preference test	Preference ratio	ns (*p* = 0.47)	ns (*p* = 0.64)
Sucrose preference test (5%, 10%, 20%)	Preference ratio	ns (*p* = 0.56)	ns (*p* = 0.11)
Prepulse inhibition (PPI) test	Percent PPI	ns (*p* = 0.85)	ns (*p* = 0.57)
	Startle magnitude	*p* < 0.005 Males > Females	ns (*p* = 0.34)

**TABLE 4:** Effect of sex and housing on body composition and high fat diet intake.

Task	Measure	Sex effect	Housing effect
2‐month weight gain	Percent gained	*p* < 0.01 Males > Females	ns (*p* = 0.44)
High fat diet choice 2‐h and 23‐h preference	Preference ratio	ns (*p* = 0.46)	ns (*p* = 0.23)
Fat pad (% body weight)	Gonadal	*p* < 0.01 Males < Females	ns (*p* = 0.79)
	Mesenteric	*p* < 0.001 Males < Females	ns (*p* = 0.79)
	Retroperitoneal	*p <* 0.05 Males > Females	ns (*p* = 0.90)

## 4 DISCUSSION

To determine whether social isolation would be detrimental in middle age, we socially isolated previously group‐housed male and female rats for 2 months and assessed their executive and metabolic function through a variety of behavioral tasks and glucose and insulin tolerance tests. Consistent with our hypothesis, socially isolated rats exhibited increased IC behavior compared to group‐housed rats. Regardless of housing condition, females exhibited more IC behavior than males. Socially isolated rats did not show increased anxiety‐like behavior, but females exhibited less anxiety‐like behavior compared to males based on the results of the NSF test. Among all groups, female socially isolated rats had the lowest blood glucose levels during the OGTT. Although housing did not affect plasma insulin concentration during the OGTT, males had higher insulin concentrations than females, indicating potential insulin resistance in male rats. This sex difference in insulin function was further supported by the higher HOMA‐IR and lower ISI_0,120_ index in male than in female rats. All rats had comparable reductions in blood glucose levels following a Humulin injection during the ITT. These findings suggest that social isolation in middle‐aged rats increases impulsive‐like behavior without affecting anxiety‐like behaviors. They also indicate that middle‐aged male rats may be more susceptible to insulin resistance than female rats, regardless of housing conditions.

### 4.1 Impulsive choice

Studies have found that impulsivity is a multifaceted construct, often divided into two differing forms of impulsive behavior: IC and impulsive action (Cho et al., 2018; Van Den Bergh et al., 2005; Wang et al., 2016). In the rodent literature, there are mixed findings on the role of social isolation on impulsivity. One study found that rats reared in social isolation beginning on postnatal day 21–23 for 12 weeks displayed greater impulsive action but less IC behavior (Liu et al., 2017). A separate study found that rats reared in isolation from postnatal day 21 for 12 weeks displayed no differences in impulsive action, but decreased IC compared to rats reared in pair housing or environmentally enriched conditions (Hellemans et al., 2005). Another study found that rats socially isolated from postnatal day 22–12 weeks of age also exhibited greater impulsive action but no change in IC behavior (Baarendse et al., 2013). However, in line with our results, two studies found that rats reared in isolation from postnatal day 21 and tested on postnatal day 69 displayed increased IC behavior compared to group‐housed animals (Kirkpatrick et al., 2013, 2014).

The discrepancy in these results has been attributed to differences in the durations spent in the isolated condition, with assessments of IC ranging from 20 days to 3 months after group or isolated housing. Additionally, some studies housed isolated rats in individual cages with regular handling, whereas others minimized handling to as little as possible. Lastly, socially isolated rats are compared with group‐housed rats that differ in the level of environmental enrichment. Some studies, including ours, provide enrichment solely through group housing. However, other studies incorporate additional forms of environmental enrichment, such as interactive objects or larger enclosures (Liu et al., 2017). It is important to note that none of the prior studies mentioned above investigated the effect of social isolation on IC in middle‐aged rats. Consequently, it is possible that social isolation during middle age leads to more pronounced effects on IC behavior compared to isolation occurring early in development. The results from the progressive ratio task indicated that group housing may alleviate impulsive behavior by enhancing food motivation, as group‐housed rats had higher break points compared to isolated rats. This effect appeared to be driven by differences in female, but not male, groups (F‐GH vs. F‐SI, *p* = 0.08; M‐GH vs. M‐SI, *p* = 0.93), suggesting a sex difference in the mechanisms underlying the effect of social isolation on IC. More studies focusing on assessing motivation in this age group are needed to support these findings.

Differences in IC behavior based on sex/gender are well established in the literature. Most human and animal studies have found that females exhibit significantly more IC behavior compared to males (Weafer & de Wit, 2014). In rodents, this finding has mainly been observed when social isolation occurs between postnatal days 30 and 90 (Koot et al., 2009; Perry et al., 2007; Perry & Carroll, 2008). Similar findings have been observed in five‐month‐old rats (Haaren et al., 1988) and in the ~15‐month‐old rats used in this study. Accordingly, compared to males, females may exhibit increased IC behavior throughout life. The mechanisms behind these sex‐dependent effects are unclear. However, a recent study by Hernandez et al. (2020) assessing the role of gonadal hormones on IC in 13‐month‐old rats found that an ovariectomy did not affect IC behavior, but an orchiectomy led to an increase in IC behavior, suggesting that IC behavior is at least partially influenced by gonadal hormones in male rats.

### 4.2 Novelty suppressed feeding

Increased latency to eat novel food and decreased food consumption during the NSF test indicate a heightened anxiety‐like behavior (Dulawa & Hen, 2005). In contrast to previous studies that found social isolation during early life and young adulthood increased anxiety‐like behavior (Dimonte et al., 2023; Lukkes et al., 2009; Magalhães et al., 2024; Skelly et al., 2015; Wright et al., 1991.), this study did not find that social isolation increased anxiety‐like behavior in either the NSF or the open field test (Figure S3). This finding also contradicts recent studies showing that social isolation increases anxiety‐like behavior in middle‐aged mice. Specifically, isolation beginning at 4 months of age for 30 weeks increased anxiety‐like behavior in the elevated plus maze (Benfato et al., 2022), and isolation starting at 45 weeks of age for 3 weeks increased anxiety‐like behavior in the OF test (Magalhães et al., 2024).

The absence of increased anxiety‐like behavior following social isolation in middle‐aged rats in this study may be influenced by the minimal handling required during their isolation period. Although handling was kept to an absolute minimum, restricted to necessary procedures like monitoring body weight and performing cage changes, prior studies suggest that limited interaction with experimenters can impact behavior. For instance, 20‐min handling on every weekday was sufficient to significantly increase center crossings in an open field in adolescent, socially isolated rats to an extent that was higher than their unhandled, isolated, and group‐housed counterparts (Song et al., 2021). It is important to note that, in our study, daily handling never exceeded 5 min per day. While it is possible that even less than 5 min of handling may have protective effects against anxiety‐like behavior, another potential reason for the absence of high anxiety‐like behavior in isolated rats is their prior social housing. All rats in this study had been socially housed for their entire lives prior to this study. Notably, rats reared in group housing for a month and then switched to individual housing for a month exhibit less anxiety‐like behaviors on an X‐maze than rats who went through the reverse sequence of housing conditions, that is, reared in individual housing and then switched to group housing (Wright et al., 1991). Given the observed beneficial effect of early life group housing for only 1 month on anxiety‐like behavior (Wright et al., 1991), it is possible that the prolonged social interaction prior to isolation provided protection against anxiety‐like behavior in our study.

Based on previous findings of increased anxiety‐like behavior in socially isolated rodents, it was surprising that we observed an increase in IC behavior following social isolation without a corresponding increase in anxiety‐like behavior. This finding may be due to the overlapping but distinct brain regions that underlie IC versus anxiety‐like behaviors. The brain regions involved in the “anxiety network” include the anterior cingulate (ACC) and pre‐ and infralimbic sub‐cortices of the prefrontal cortex (PFC), hippocampus, amygdala, hypothalamus, striatum, and the bed nucleus of the stria terminalis (Xie et al., 2021). While the PFC is also implicated in IC behaviors, it is the orbitofrontal cortex (OFC) rather than the ACC or the pre‐ and infralimbic sub‐cortices that seem to play a key role in IC behavior. Prior studies have found that adolescent social isolation is sufficient to induce changes in the connection between the OFC and the basolateral amygdala (Kuniishi et al., 2022). Furthermore, studies have shown that social isolation can lead to a reduction in crucial inhibitory, parvalbumin‐positive interneurons in the left OFC (Jeon et al., 2023), which may alter OFC activity and influence IC behavior. In middle‐aged rats, it is possible that social isolation primarily affects the OFC without triggering the broader “anxiety network”. This selective effect on the OFC could explain the increase in IC behavior without a corresponding increase in anxiety‐like behavior, as the mechanisms driving impulsivity may operate independently of those involved in anxiety. Notably, the studies mentioned have examined the effect of social isolation in younger animals when their neural circuitry is still developing. In middle age, as neural circuits become more stable, changes in the OFC may have a more targeted impact, potentially influencing circuits associated with impulsive choice while sparing those involved in anxiety‐like behavior.

### 4.3 Metabolic function

Given findings in humans that social isolation is associated with a higher risk of developing type 2 diabetes, this study investigated the impact of social isolation on glucose metabolism and insulin sensitivity. Contrary to our hypothesis, isolated rats did not exhibit glucose intolerance compared to group‐housed rats. Two hours following the bolus of glucose, all rats had a blood glucose level below 140 mg/dL, indicating normal glucose tolerance (Jagannathan et al., 2020). However, during the OGTT, males exhibited higher insulin levels and a higher insulin AUC compared to females. Since hyperinsulinemia is believed to be a precursor to insulin resistance, and insulin resistance is a key component in the pathogenesis of type 2 diabetes (Janssen, 2021), we utilized the HOMA‐IR formula and the insulin sensitivity index (ISI_0,120_) for further analysis. All rats exhibited HOMA‐IR values exceeding the 2.6 threshold (Ascaso et al., 2003), indicating insulin resistance, with male rats exhibiting significantly higher HOMA‐IR values than females. The ISI _0,120_ values also indicated that males exhibited greater peripheral insulin resistance compared to females. These results align with human studies demonstrating that insulin resistance is more prevalent in men than in premenopausal middle‐aged women (Ciarambino et al., 2023). As female rats do not experience menopause but instead have irregular estrous cycles and low levels of ovarian hormones after middle age (Ajayi & Akhigbe, 2020), utilizing a rodent model that mimics aspects of menopause may provide more insightful data on the sex‐dependent role of social isolation in metabolic dysfunction. Given that women typically undergo menopause in middle age, such a model could better illuminate the potential metabolic impacts of social isolation during this critical life stage.

The ISI _0,120_ and HOMA‐IR results may seem counterintuitive to the findings that all rats adequately cleared glucose during OGTT and showed significant hypoglycemia during ITT. However, it is important to consider that prior to experiencing hyperglycemia, individuals often exhibit increased insulin secretion. This compensatory hyperinsulinemia can lead to insulin resistance where more insulin is required to maintain homeostasis in blood glucose levels (Ciarambino et al., 2023; Yang et al., 2016). Over time, the body becomes unable to sustain this response, resulting in elevated blood glucose levels characteristic of type 2 diabetes. The lack of apparent metabolic dysfunction following social isolation in this study contrasts with recent findings that showed male rats socially isolated from postnatal day 21–7 weeks of age had increased HOMA‐IR and changes in hypothalamic AKT and GLUT‐4 levels compared to their group‐housed counterparts (Bove et al., 2022). Specifically, socially isolated rats had decreased hypothalamic AKT phosphorylation and GLUT‐4 levels. GLUT‐4 is an insulin‐dependent transporter crucial for glucose uptake and utilization. The AKT pathway induces the translocation of GLUT‐4 to the neuronal cell membrane, enhancing transport during periods of high metabolic demand (Bove et al., 2022). Therefore, these findings indicate that social isolation can disrupt both peripheral and central insulin signaling.

Following an episode of hypoglycemia, a counterregulatory response is initiated. This involves activation of the sympathetic nervous system and the release of glucagon, adrenaline, and corticosterone to restore blood glucose to homeostatic levels (Durham & Truett, 2006; Verberne et al., 2014; Yang et al., 2020). Therefore, a slow rise in blood glucose levels following the hypoglycemia during ITT may indicate a deficiency in this counterregulatory response. Although peripheral insulin is crucial for lowering blood glucose levels, central insulin is also involved in the counterregulatory response to hypoglycemia. This is evidenced by the finding that adult mice with central insulin receptor knockout show a reduced counterregulatory response to hypoglycemia compared to wild‐type mice (Agrawal et al., 2021; Fisher et al., 2005). Notably, female socially isolated rats in our study had lower blood glucose levels than female group‐housed rats at 90 and 240 min post‐Humulin injection (Figure S9A; post hoc *p* < 0.05), suggesting a delayed counterregulatory response to recover from the Humulin‐induced hypoglycemia. This implies that social isolation in female rats may lead to dysfunction in the central insulin signaling that can facilitate a recovery of blood glucose levels following hypoglycemia. Additionally, the AUC of raw blood glucose levels during the ITT (Figure S9B) revealed that F‐SI rats had significantly lower blood glucose AUC compared to M‐SI rats, M‐GH rats, and F‐GH rats (post hoc *p* < 0.05), providing further evidence of a delayed counterregulatory response in female socially isolated middle age rats. However, this effect was not observed when data was normalized, indicating that it is a marginal effect. In future studies, we aim to investigate the central insulin signaling pathway to better assess whether social isolation affects central insulin function.

## 5 CONCLUSION

The effects of social isolation during later stages of life are not researched sufficiently. This study aimed to fill this gap by investigating the impact of social isolation on middle‐aged rats. The sex‐dependent results underscore the complex effects of social isolation on metabolic and behavioral functions and highlight the importance of considering sex‐specific responses in future research. Additionally, in women and female rodents, changes in menstrual and estrous cycling occur during middle age. Therefore, future studies would benefit from investigating whether the changes in behavior and metabolic responses seen following social isolation may be mediated differently by different phases of the ovarian hormonal cycle in females. In humans, mid‐ to late‐life impulsivity is associated with an increased risk of dementia, and it has been suggested that increased impulsivity may be an early indicator of Alzheimer's disease pathology in healthy individuals (Bateman et al., 2020). Increased IC specifically has been associated with other negative health outcomes such as an increased risk of relapse in individuals with substance use disorders (Stevens et al., 2015). Therefore, it is important to understand the influence of isolation on perpetuating impulsive behavior. Identifying and addressing the factors that contribute to increased IC can provide insight for developing preventive strategies and interventions to mitigate these negative health outcomes, ultimately improving the overall quality of life of individuals vulnerable to experiencing social isolation. Although our study did not find an effect of social isolation on metabolic function, future research should investigate the combined effect of prolonged Western diet consumption and social isolation. The palatable, energy‐dense Western diet is typically consumed throughout the lifespan and considered as comfort food to cope with stress such as social isolation in modern humans. Therefore, a prolonged exposure to the Western diet is more representative of actual consumption compared to a short‐term exposure. Subsequent investigation on the extent to which prolonged consumption of a Western diet in combination with social isolation can facilitate metabolic dysfunction in the rodent model presented here may provide further insights into the role of social isolation on promoting the development and prognosis of type 2 diabetes.

## FUNDING INFORMATION

This study was supported by intramural funding within UIUC and no extramural funding was utilized.

## CONFLICT OF INTEREST STATEMENT

The authors have no conflicts of interest.

## ETHICS STATEMENT

The experimental protocol was approved by an Institutional Review Committee for the use of Animal Subjects and the procedures are in compliance with the National Institutes of Health Guide for Care and Use of Laboratory Animals.

## COVER NOTE

We would like to transfer this manuscript that reports the influences of social isolation on executive and metabolic function in middle‐aged male and female rats from AJP to Physiological Reports. The research is original and has not been published or submitted for publication elsewhere while under consideration of this journal. Part of the results was presented at the annual meeting of the Society for the Study of Ingestive Behavior in 2023. Furthermore, there are no competing financial interests in relation to the research described. Methods are detailed in the manuscript, and raw data will be available upon request.

## Supporting information


Appendix S1.

## Data Availability

Raw data will be available upon request.
