# Correction to “Social Isolation Increases Impulsive Choice With Minor Changes on Metabolic Function in Middle‐Aged Rats”Venegas, J. J., J. M. Weisz, C. Y. Choi, R. E. Herringshaw, O. A. Nabelsi, and N.‐C. Liang. 2025. “Social Isolation Increases Impulsive Choice With Minor Charges on Metabolic Function in Middle‐Aged Rats.” Physiological Reports 13, no. 2: e70184 https://doi.org/10.14814/phy2.70184.

**DOI:** 10.14814/phy2.70974

**Published:** 2026-06-14

The authors regret some text errors in the published paper.

In 2.6 of “MATERIALS AND METHODS”, the formula for the insulin sensitivity index (ISI_0,120_) was incorrect

$${\mathrm{ISI}}_{0,120}=\frac{\text{glucose load}\;\left(\mathrm{mg}\right)+\left({\text{glucose}}_{0\min}-{\text{glucose}}_{120\min}\left(\frac{\mathrm{mg}}{L}\right)\right)\times 0.19\text{body weight}\;\left(\mathrm{kg}\right)}{120\times \log\left(\frac{{\text{insulin}}_{0\min}-{\text{insulin}}_{120\min}\left(\frac{\mathrm{mU}}{L}\right)}{2}\right)+\left(\frac{{\text{glucose}}_{0\min}-{\text{glucose}}_{120\min}\left(\frac{\text{mmol}}{L}\right)}{2}\right)}$$

The formula should have been

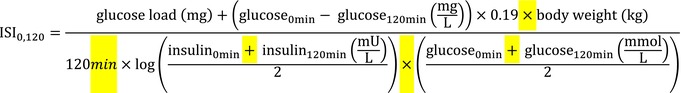

The highlighted areas show where the errors occurred. We confirmed that calculations were done using the correct formula, so the results of ISI_0,120_ reported in the manuscript were correct.

In 3.6 of “RESULTS”, the text “A two‐way ANOVA on the AUC revealed no housing [*F*(1, 37) = 3.50, *p* = 0.07; Figure 6b], sex [*F*(1, 37) = 0.15, *p* = 0.70], or interaction effects [*F*(1, 37) = 2.97, *p* = 0.09].” was inaccurate. This should have read “A two‐way ANOVA on the AUC revealed no housing [*F*(1, 37) = 3.30, *p* = 0.08; Figure 6b], sex [*F*(1, 37) = 0.14, *p* = 0.71], or interaction effects [*F*(1, 37) = 2.68, *p* = 0.11].” Original blood glucose AUC during the insulin tolerance test reported was calculated by an inaccurate formula, which we identified while conducting another study recently. The data in Panel a, Insulin Tolerance Test, are unchanged.

Although AUC calculated from the correct formula does not change the results and conclusion, we would like to transparently report our mistakes and provide the most accurate information for the readers. Accordingly, Figure 6 should be updated as below where the AUC bar graph includes the numbers calculated by the correct formula.

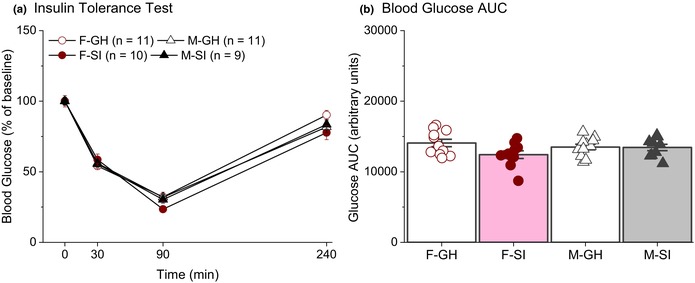

In “Insulin Tolerance Test – analysis of raw data” of Supplementary Materials, the text “A two‐way ANOVA on the blood glucose AUC revealed no main effect of housing [*F*(1,37) = 1.94, *p* = 0.17; Figure S9b]. However, there was a significant main effect of sex [*F*(1,37) = 12.29, *p* < 0.01], and sex by housing effect [*F*(1,37) = 8.11, *p* < 0.01]” was inaccurate. This should have read “A two‐way ANOVA on the blood glucose AUC revealed no main effect of housing [*F*(1,37) = 1.86, *p* = 0.18; Figure S9b]. However, there was a significant main effect of sex [*F*(1,37) = 11.08, *p* < 0.01], and sex by housing effect [*F*(1,37) = 7.30, *p* < 0.02].” Original blood glucose AUC during the insulin tolerance test reported was calculated by an inaccurate formula. The AUC calculated from the correct formula does not change the results, statistical results or conclusion. However, Figure S9 should be updated as below where the AUC bar graph includes the numbers calculated by the correct formula. Data on Panel a, Insulin Tolerance Test, are unchanged.

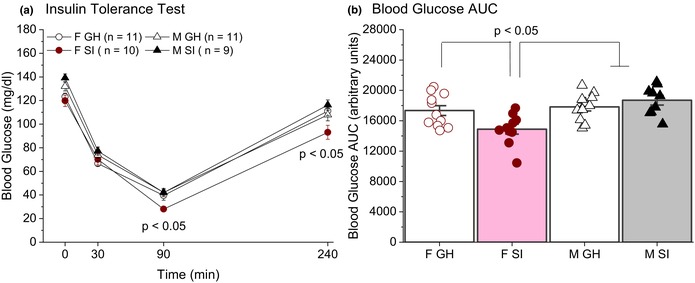

We apologize for these errors, which do not affect the scientific conclusions of the study.

