# Supplementary material for: Social isolation increases impulsive choice with minor changes on metabolic function in middle‐aged rats
Source: Physiol Rep. 2025 Jan 16;13(2):e70184. doi: 10.14814/phy2.70184 (PMC11738651; doi:10.14814/phy2.70184)
Supplement: Supplementary file 1 — Appendix S1. [file PHY2-13-e70184-s001.docx]

**Supplemental methods with results not included in the manuscript**

**Figure S1.** Experimental Timeline. Tasks highlighted in yellow are discussed in this document, while non-highlighted tasks are included in the main text of the manuscript. Seven days after arrival, rats underwent a fasting blood glucose (FBG) test. Two days later, they were either group housed (GH) or socially isolated (SI). At 13 months of age, behavioral testing began.


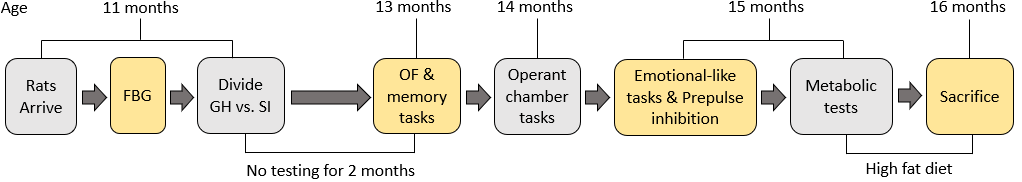


*Data Analysis*

Statistical analysis was conducted in RStudio (version 4.2.1). Two-way ANOVAs were performed with sex and housing as between-subject factors, and Tukey’s HSD post hoc analysis was applied for significant main effects. Kruskal-Wallis test were implemented when two-way ANOVA assumptions were violated. Two Separate three-way mixed ANOVAs were employed to analyze escape latency and errors in the Barnes maze across trials (1 - 4) and reversal trials (R1 - R3). Trials served as the within-subject factor, while sex and housing were between-subject factors. Additionally, separate three-way mixed model ANOVAs were utilized to assess sucrose preference, startle magnitude, percent prepulse inhibition, and raw insulin tolerance test results. In each of these three-way mixed model ANOVAs, sex and housing were considered between-subject variables, and the repeated measure (such as sucrose concentration, decibel, block, or time) served as within-subject variables. If sphericity assumptions were violated, a Greenhouse-Geisser was applied.

*Fasting Blood Glucose*

Seven days after the rat’s arrival, and before being assigned to either socially isolated (SI) or group housed (GH) conditions, all rats underwent a fasting blood glucose (FBG) test. On the day of testing, rats were individually placed in holding cages at 0700 h with access to water but no food. Two hours after light onset (1500 h), rats were moved into the testing room and allowed to habituate for 30 minutes. Fasting blood glucose levels were measured using an AlphaTRAK2 glucometer, and tail blood samples were collected.

A two-way ANOVA was conducted to detect differences in blood glucose levels across the groups. There were no housing [F(1,40) = 1.66, p = 0.21; Figure S2A] or interaction effects [F(1,40) = 0.28, p = 0.60]. However, females exhibited a higher fasting blood glucose than males [F(1,40) = 6.74, p = 0.01].

**Figure S2**. **(A)** Prior to placement in socially isolated (SI) or group housing (GH), females had higher FBG levels compared to males. **(B)** After two months in respective housing condition, males gained significantly more weight than females.

*Percent Weight Gain*

Percent weight gain was calculated by subtracting the initial weight of the rats from their weight after 2 months in their assigned housing conditions. Two separate Kruskal-Wallis tests were conducted to assess the effect of sex or housing on percent weight gained. There was no effect of housing [H(1) = 0.61, p = 0.44; Figure S2B]. However, there was a significant effect of sex [H(1) = 8.13, p < 0.01]. Females gained less weight than males.

*Open Field Test*

The open field arena measured 25 x 18” and was divided into central (18 x 11”) and peripheral areas. Rats were placed in individual holding cages two hours after light onset, transported to the testing room, and allowed to habituate for 30 minutes. Subsequently, each rat was placed in the center of the open field arena and given 5 minutes to explore. The arena was cleaned between each rat’s session to remove olfactory cues. The open field test was recorded for later scoring of the total time spent in the center versus peripheral areas and the total number of entries into the center.

Center time and center entries were assessed using two separate two-way ANOVAs. There was no main effect of housing on center time [F(1,40) = 1.91, p = 0.18; Figure S3A] or on center entries [F(1,40) = 0.28, p = 0.60; Figure S3B]. There was a main effect of sex on center time [F(1,40) = 7.88, p < 0.01]. Males spent more time in the center than females. This effect was not observed in entries, as both male and female rats made comparable center entries [F(1,40) = 0.54, p = 0.47].

**Figure S3.** Open field results. Housing had no effect on anxiety-like behaviors. Socially isolated and group housed rats spent comparable **(A)** time in the center and made similar **(B)** center entries. **(A)** Males spent significantly longer in the center compared to female rats.

*Object Location, and Novel Object Recognition*

The day after the open field test, rats were placed in the same arena to undergo a familiarization session. During the familiarization session, two identical objects were positioned in the center of the arena, and rats were given 5 minutes to explore. The exploration time for each object was later assessed from video recordings. For this and subsequent sessions, the arena and objects were cleaned between rats to prevent odor cues from influencing exploration time.

The following day, all rats participated in an object location (OL) test in the same arena. The identical objects from the familiarization session were used, except one object was moved to a new location. This object's placement was counterbalanced among the rats, and each was allowed 5 minutes of exploration. Video recordings were later analyzed to assess the time spent exploring the object in both the original and novel locations.

The next day, a novel object recognition (NOR) test was conducted. The object that had not been moved previously was replaced with a novel object of a similar size but differing in color and shape. Like the OL test, rats were given 5 minutes to explore, and video analysis was used to determine the time spent exploring each object.

**Figure S4.** Object location and novel object recognition results. **(A)** Housing and sex had no effect on the discrimination index. **(B)** Regardless of housing, females had a higher discrimination index than males. Note: on average, in either task, no animals performed above chance (0.50).

To assess spatial memory, a two-way ANOVA was conducted on the discrimination index of the OL. The OL discrimination index was calculated as time spent exploring the object in the novel location minus time spent exploring the object in the familiar location, divided by total time spent exploring objects in both locations. There was no sex [F(1,40) = 2.77, p = 0.10; Figure S4A], housing [F(1,40) = 0.01, p = 0.91], or interaction effects [F(1,40) = 0.31, p = 0.58] on the discrimination index. It is important to note that most rats spent an equivalent amount of time exploring objects in both locations, as evidenced by a discrimination value of 0.00. This suggests that the rats did not differentiate between the two locations.

The NOR task was employed to evaluate recognition memory across groups. A two-way ANOVA was performed on the discrimination index. Similar to the OL task, the NOR discrimination index was computed by dividing the total time spent exploring the novel object – total time spent exploring the familiar object by the total time spent exploring both objects. Regardless of housing [F(1,40) = 0.02, p = 0.90; Figure S4B], females exhibited a higher discrimination index than males [F(1,40) = 4.45, p = 0.04]. However, as observed in the OL task, none of the rats performed above chance (0.50), indicating that they did not differentiate between the two objects.

*Barnes maze*

At approximately 13 months of age all rats underwent training and testing on a Barnes maze, which began 2.5 hours (1630 h) after light onset and ended no later than 3.5 hours (2230 h) before dark onset. The maze was 122 cm in diameter placed on a 360° rotating system. Along the edge of the circular maze, there were 20 holes that were 10 cm in diameter. Underneath one of the 20 holes there was an escape box equipped with a ramp. Rats learned to use the visual cues in the testing room to locate the escape box through the training days. After each trial the maze and escape box were cleaned with a non-alcohol disinfectant and deodorizer solution. Each trial was recorded using a concealed ceiling mounted camera that captured the entire maze.

Following daily body weight measurements, all rats were individually placed in holding cages and allowed to habituate in the room adjacent to where testing occurred. The testing order was counterbalanced to avoid any effect of time of day on performance. Rats underwent two trials a day across eight days during training, i.e., a total of 16 trials. During training trials, rats were placed on one of the four starting locations facing away from the center of the maze. Two starting positions were assigned for training on odd days and the other two starting positions for training on even days. Rats had 90 seconds to explore the maze and find the escape box. If they did not find the escape box within that time, they were guided to the box by the researcher. Once the rat entered the escape box, it was covered, and the rat remained there for 30 seconds before being returned to its holding cage. All rats completed the first trial before beginning the second trial.

Testing began on the ninth day after 8 days of training. Day nine consisted of the probe (P) and reversal one (R1) trial. During the probe trial rats were placed in the center of the maze facing away from the escape box. The procedures were identical to the training trials. In the R1 trial the maze was rotated 180° so that the escape box would be on the opposite side; the starting position was the same as in the probe trial. On day 10, rats underwent two more reversal trials with two different starting positions (R2 & R3). During reversal trials, rats were given 150 seconds to find the escape box. If the rat failed to find the escape box, it would be gently guided to it and allowed to remain in the box for 30 seconds.

Performance during training and testing days were manually scored for latency and errors committed before entering the escape box. Latency was the amount of time it took the rat to find the escape box after being placed on the maze. An error was counted any time a rat submerged its head through a hole that was not the escape box. Video scoring of training and testing trials were completed by two researchers.

All rats completed four trials for each starting position across eight days with two training trials starting from two different positions on each day. For the analysis, the 16 trials across 4 locations were categorized such that trial 1 (T1) represented the four training trials on days 1 and 2, trial 2 (T2) represented the four training trials on days 3 and 4, trial 3 (T3) represented the four training trials on days 5 and 6, and trial 4 (T4) represented the four training trials on days 7 and 8. One rat was excluded from the analysis because it was placed on an incorrect starting position during one of the trials. During T1 to T4 there was no interaction effect between sex, housing and trial on escape latency [F(3,117) = 0.78, p = 0.51; Figure S5A] or errors [F(3,117) = 1.51, p = 0.22; Figure S5B]. A significant effect of trial on escape latency [trial F(3,117) = 0.93, p < 0.001] and errors [trial F(3,117) = 50.31, p < 0.001] indicated that the rats acquired the Barnes maze task. Two separate two-way ANOVAs were conducted on escape latency and errors during the probe trial. There was no significant effect of housing [F(1,39) = 0.07, p = 0.79; Figure S5A] or sex [F(1,39) = 1.81, p = 0.19] on escape latency. There was also no effect of housing [F(1,39) = 0.44, p = 0.51; Figure S5B] or sex [F(1,39) = 1.82, p = 0.19] on errors made. A three-way mixed model ANOVA on the data collected during the reversal trials revealed no significant main or interaction effects on escape latency or errors.

**Figure S5.** Barnes maze results. Socially isolated and group housed male and female rats exhibited comparable **(A)** escape latencies and **(B)** errors across training, probe, and reversal trials.

*Sucrose Preference Test*

During the first day of testing, all rats were placed in individual holding cages with food and access to two bottles: one containing a 1% sucrose solution and the other filled with tap water. Testing began two hours after light onset (1600 h) for a total duration of 23 hours. To prevent side preference, the bottles were switched 12 hours into the test. Following the 23-hour period, total intake of sucrose and water in grams was recorded.

Following the 23-hour sucrose preference test (SPT), rats underwent three separate one hour SPTs over the course of three days, with sucrose concentrations increasing daily from 5% to 10% to 20%. During each session, rats were given access to a bottle of water and a bottle of the sucrose solution without any food provided. The placement of the solutions was counterbalanced. Total intake in grams of each solution was recorded for each session.

Sucrose preference was calculated as the total sucrose consumed divided by total fluid intake. Results from the 23-hour SPT revealed no main effects of sex [F(1,40) = 0.53, p = 0.47], housing [F(1,40) = 0.23, p = 0.64], or interaction effects observed (Figure S6A). Following the 23-hour 1% SPT, rats underwent a 1-hour SPT over three days with an escalating dose of the sucrose solution. A three-way mixed model ANOVA was conducted to assess sucrose preference across concentrations. Similarly to the 23-hour SPT, there were no main effects of sex [F(1,40) = 0.34, p = 0.56], housing [F(1,40) = 2.70, p = 0.11], or interaction effects on the 1-hour SPT (Figure S6B).

**Figure S6.** Results of the sucrose preference tests. **(A)** Depression-like behavior was assessed by exposing rats to water and a 1% sucrose solution over 23 hours. All rats preferred the 1% sucrose solution over water. **(B)** Regardless of sex and housing, all rats exhibited a similar preference for the different concentrations of sucrose solution.

*Prepulse Inhibition*

Deficits in sensory motor gating can be assessed using the prepulse inhibition (PPI) task, where a loud startle response is reduced if preceded by a quieter prepulse. Changes in acoustic startle response serve as a measure of sensorimotor gating efficiency. A decrease in startle magnitude when a prepulse is present indicates better sensorimotor gating. To assess PPI, the SR-LAB™ Startle Response System from San Diego Instruments (San Diego, CA) was used. This system consists of a cabinet with a plexiglass cylinder in the center mounted onto a platform capable of detecting the rats' startle response through changes in their movement when a pulse is presented. This response is converted into data using SR lab software version 96.1.5.66.

On the first day, two hours after light onset (1600 h), rats were individually placed in their holding cages and transported to the waiting room where they habituated for 30 minutes. Following habituation, rats underwent the acclimation session where they were placed in the plexiglass cylinder and exposed to background noise at 65 decibels (dB) for 10 minutes. The following day, startle magnitude was assessed using the procedure previously described by Sellinger et al. (2020).

To assess startle magnitude across blocks, a three-way mixed model ANOVA was conducted. Due to a violation of the sphericity assumption, the three-way mixed model ANOVA was conducted with a Greenhouse-Geisser correction. There was a significant effect of startle magnitude across blocks [F(2.38, 85.81) = 12.04, p < 0.001; Figure S7A]. There was also a significant main effect of sex [F(1,36) = 17. 21, p < 0.001] and interaction effect of sex by block [F(2.38, 85.81) = 4.02, p < 0.05]. Male rats had a greater startle magnitude across prepulse intensity compared to females. However, there was no main effect of housing [F(1,36) = 0.95, p = 0.34] or interaction effect of housing by block [F(2.38, 85.81) = 1.65, p = 0.19].

Percent prepulse inhibition (PPI) was calculated as pulse alone minus the prepulse plus pulse divided by the pulse alone times 100. A three-way mixed model ANOVA was implemented to assess the change in percent PPI across prepulse intensity. As expected, rats exhibited a higher percent PPI as the prepulse intensity increased [F(2,72) = 78.75, p < 0.001; Figure S7B], indicating a decreased startle response. There was no main effect of sex [F(1,36) = 0.11, p = 0.74] or housing [F(1,36) = 2.70, p = 0.11. There was no interaction effect of housing by prepulse intensity [F(2,72) = 1.11, p = 0.34]

**Figure S7.** Prepulse inhibition (PPI) results. **(A)** Across blocks, females exhibited a lower startle magnitude following a prepulse compared to male rats. **(B)** All rats exhibited similar % PPI across different prepulse intensity, indicating comparable sensorimotor gating abilities.


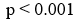


*High Fat Diet choice*

To assess changes in diet preference, rats underwent a high fat diet (HFD) choice test. During testing, rats were individually housed in standard cages with access to water and two types of food; standard chow (3.1 kcal/g; Envigo Teklad 18% protein diet), and the high fat Western diet (4.7 kcal/g; 43% carbohydrates, 40% fat, 17% protein; D12079B, Research Diets, New Brunswick, NJ). The rats remained in their individual cages for a 23-hour period. The total amount of food consumed of each diet was recorded in grams.

HFD preference was calculated as total calorie consumed of the high fat diet divided by total calorie consumed of both diets. Separate Kruskal-Wallis tests were conducted to assess HFD preference over 2 and 23 hours. After two hours, there was no main effect of sex [H(1) = 1.16, p = 0.28; Figure S8A] or housing [H(1) = 0.003, p = 0.96]. All rats preferred the HFD. Similar results were seen at 23 hours after the HFD choice test; there was no effect of sex [H(1) = 2.92, p = 0.09; Figure S8B] or housing [H(1) = 1.65, p = 0.20]. All rats continued to prefer the HFD.

**Figure S8.** High fat diet (HFD) choice preference results. **(A, B)** Regardless of housing or sex, all rats preferred the HFD to the standard chow.

*Insulin Tolerance Test – analysis of raw data*

A three-way mixed model ANOVA was conducted with the raw blood glucose levels, e.g., non-normalized data, during the insulin tolerance test. Rats that were excluded in the normalized data analysis were excluded here. Unlike the negative findings from the normalized data, there was a main effect of sex [F(1,37) = 14.73, p < 0.005; Figure S9A]. Females had lower blood glucose levels than males. Additionally, there was a sex by housing interaction effect [F(1,37) = 5.49, p = 0.03]. F-SI rats exhibited a lower blood glucose level compared to F-GH rats at the 90- and 240-minute time point (post hoc p < 0.05) indicating a delay in the counterregulatory mechanism to hypoglycemia. Similarly to the normalized data, there was no main effect of housing [F(1,37) = 0.28, p = 0.60].

A two-way ANOVA on the blood glucose AUC revealed no main effect of housing [F(1,37) = 1.94, p = 0.17; Figure S9B]. However there was a significant main effect of sex [F(1,37) = 12.29, p < 0.01], and sex by housing effect [F(1,37) = 8.11, p < 0.01]. Females had a lower blood glucose level than males. F-SI rats had a lower blood glucose AUC compared to F-GH, M-SI and M-GH, providing evidence for a reduced counterregulatory response (post hoc both p < 0.05)

**Figure S9.** Results of non-normalized blood glucose levels during the insulin tolerance test. (A) All rats showed a reduction in blood glucose levels 30 minutes post insulin injection. F-SI rats displayed a delay in the counterregulatory response to hypoglycemia, assessed at the 90-minute time point. F-SI rats had significantly lower blood glucose levels than F-GH rats 90 and 240 minutes post injection. (B) F-SI rats had a lower blood glucose AUC than M-SI, M-GH, and F-GH rats, indicating a reduced counterregulatory response to hypoglycemia.

*Fat Pad Dissection*

The carcasses were stored in a -20℃ freezer following sacrifice. Prior to tissue dissection, the bodies were defrosted for 42 hours in a 4℃ fridge. An incision was made from the lower abdomen to the chest plate, extending into the thoracic cavity by cutting the skin and ribs from the sternum to the armpit. This allowed for full visualization of the peritoneal cavity. Fat was carefully collected from the gonadal, mesenteric, and retroperitoneal region and weight was measured in grams.

Three separate two-way ANOVAs were conducted to assess the fat composition (g/100 g of body weight) per region. All regions showed no effect of housing or sex by housing interaction. There was a significant main effect of sex per each region. Females had greater gonadal [F(1,39) = 105.21, p < 0.001; Figure S10 and mesenteric [F(1,39) = 15.37, p < 0.001] fat compared to males. Males had more retroperitoneal fat than females [F(1,39) = 7.52, p < 0.01].

**Figure S10.** Results of visceral fat composition. Females exhibited greater compositions of gonadal and mesenteric fat compared to males, while males had higher retroperitoneal fat compared to females.

References

Sellinger EP, Kougias DG, Drzewiecki CM, Juraska JM. Behavioral effects in adult rats exposed to low doses of a phthalate mixture during the perinatal or adolescent period. Neurotoxicol Teratol. 2020 May-Jun;79:106886. doi: 10.1016/j.ntt.2020.106886. Epub 2020 Apr 18. PMID: 32315766; PMCID: PMC7260060.
